# Modulatory mechanisms and multiple functions of somatodendritic A-type K^**+**^ channel auxiliary subunits

**DOI:** 10.3389/fncel.2014.00082

**Published:** 2014-03-27

**Authors:** Henry H. Jerng, Paul J. Pfaffinger

**Affiliations:** Department of Neuroscience, Baylor College of MedicineHouston, TX, USA

**Keywords:** somatodendritic A-type current, potassium channel, auxiliary subunit, Kv channel-interacting protein, dipeptidyl peptidase-like protein, N-terminal variant, modulatory mechanism, excitability

## Abstract

Auxiliary subunits are non-conducting, modulatory components of the multi-protein ion channel complexes that underlie normal neuronal signaling. They interact with the pore-forming α-subunits to modulate surface distribution, ion conductance, and channel gating properties. For the somatodendritic subthreshold A-type potassium (I_SA_) channel based on Kv4 α-subunits, two types of auxiliary subunits have been extensively studied: Kv channel-interacting proteins (KChIPs) and dipeptidyl peptidase-like proteins (DPLPs). KChIPs are cytoplasmic calcium-binding proteins that interact with intracellular portions of the Kv4 subunits, whereas DPLPs are type II transmembrane proteins that associate with the Kv4 channel core. Both KChIPs and DPLPs genes contain multiple start sites that are used by various neuronal populations to drive the differential expression of functionally distinct N-terminal variants. In turn, these N-terminal variants generate tremendous functional diversity across the nervous system. Here, we focus our review on (1) the molecular mechanism underlying the unique properties of different N-terminal variants, (2) the shaping of native I_SA_ properties by the concerted actions of KChIPs and DPLP variants, and (3) the surprising ways that KChIPs and DPLPs coordinate the activity of multiple channels to fine-tune neuronal excitability. Unlocking the unique contributions of different auxiliary subunit N-terminal variants may provide an important opportunity to develop novel targeted therapeutics to treat numerous neurological disorders.

## INTRODUCTION TO I_SA_

The membranes of neurons contain multiple sets of voltage-sensitive K^+^ (Kv) channels that open and close in response to changes in membrane potential, giving rise to outward K^+^ currents with differing voltage dependence, kinetic properties, and pharmacological sensitivities. One distinct K^+^ current, the subthreshold A-type transient current (I_SA_), has garnered a great deal of interest since its initial discovery in the neuronal somata by Hagiwara et al. (1961). Later characterized in detail by Connor and Stevens (1971b) and Neher (1971), I_SA_ activates rapidly in the subthreshold range of membrane potentials but rapidly inactivates. At resting membrane potentials more positive than around -50 mV, no I_SA_ current can be elicited unless the membrane is first conditioned to more hyperpolarized potentials to allow recovery from inactivation (Connor and Stevens, 1971a, b; Neher, 1971). Pharmacologically, I_SA _is identifiable by its elevated sensitivity to block by 4-aminopyridine and considerably reduced sensitivity to tetraethylammonium compared to most other Kv channel currents (Thompson, 1977). Currents with properties matching I_SA_ are found in nearly all excitable cells, both neuronal and non-neuronal (Rudy, 1988).

Studies on I_SA_ functional properties and subcellular localization in neurons have suggested important roles for I_SA_ in neurophysiology. In repetitively firing cells, an inactivation-and-recovery cycle for I_SA_ during the interspike interval regulates spiking timing and firing frequency (Connor and Stevens, 1971a). Because of its rapid activation, I_SA_ is also capable of suppressing synaptic excitatory potentials and delaying spike firing in response to a superthreshold stimulus (Schoppa and Westbrook, 1999; Shibata et al., 2000; Nadin and Pfaffinger, 2010). In the late 1990’s, it was discovered that I_SA_ has an important role in regulating signaling thought to be critical for Hebbian plasticity. In hippocampal pyramidal neurons, in addition to firing an all-or-nothing orthodromic spike down the axon, a graded spike is backpropagated up the dendritic tree. Due to its high density in the dendrites and its rapid activation, I_SA_ suppresses action potential backpropagation (Hoffman et al., 1997). However, in regions of the dendritic tree that experienced recent depolarizing synaptic activity sufficient to inactivate I_SA_, the backpropagating action potential remains large and induces a large influx of Ca^2^^+^ that is critical for long-term potentiation (LTP; Johnston et al., 2003). The ability of I_SA_ to compartmentalize excitability and information storage in the dendrites may have been a critical factor in the evolution of the nervous system.

## THE I_SA_ CHANNEL IS A MULTI-PROTEIN SUPERMOLECULAR COMPLEX

In the 1980s, genetic analysis of the fruit fly *Drosophila melanogaster* provided an opportunity to probe the molecular basis of I_SA_. The identification of the *Shaker* gene mutants led to the discovery of the first voltage-gated K^+^ channel, Shaker, and the identification of other subfamilies of Kv channels in the fly (Shab, Shaw, and Shal) as well as their mammalian homologs (Kv1, Kv2, Kv3, and Kv4; Papazian et al., 1987; Pongs et al., 1988; Butler et al., 1989; Chandy and Gutman, 1993). Of these initial four subfamilies of cloned Kv channels, Kv4 was identified as the best candidate for underlying I_SA_ since Kv4 channels show localization in the soma and dendrites (Sheng et al., 1992) and exhibit biophysical and pharmacological properties that most resemble native I_SA_ in heterologous expression systems (Jerng et al., 2004a). Subsequent breakthrough studies examining the native I_SA_ using genetic knockouts, dominant negative suppression, and RNA interference (RNAi) techniques confirm that indeed Kv4 channels underlie I_SA_ (Johns et al., 1997; Malin and Nerbonne, 2000; Chen et al., 2006b; Lauver et al., 2006).

Although Kv4 subunits underlie I_SA_, the functional properties of Kv4 channels in heterologous cells differ from those of native I_SA_, especially the kinetics of activation, inactivation and recovery from inactivation (Serodio et al., 1994). As accumulating research began to show that ion channels and receptors function as large macromolecular complexes, An et al. (2000) discovered the first Kv4 auxiliary subunit by using yeast two-hybrid screens with a Kv4 N-terminal domain as bait. Named Kv channel-interacting proteins, KChIPs are cytoplasmic Ca^2^^+^-binding proteins that significantly increased surface expression of Kv4 current and remodeled current biophysical properties. However, reconstitution studies in heterologous cells showed that co-expression of KChIP with Kv4 channels alone is insufficient to generate the native I_SA_. Especially critical is the failure of KChIPs to recapitulate the characteristic features of I_SA_: rapid kinetics of activation, inactivation, and recovery from inactivation.

In the search for additional Kv4 interacting proteins, the discovery of KChIP was quickly followed by another Kv4 modulatory protein, the dipeptidyl peptidase-like proteins (DPLPs). DPLPs are single-pass type II transmembrane proteins belonging to the S9B family of serine proteases, and they consist of DPP6 (aka DPPX) and DPP10 (aka DPPY; Wada et al., 1992; Qi et al., 2003; Takimoto et al., 2006). Although DPLPs are closely related to the DPP4 family of serine proteases, they lack both the active site serine and the molecular architecture necessary for catalytic activity (Kin et al., 2001; Strop et al., 2004). Instead of proteolytic activity, DPP6 and DPP10 interact with Kv4 proteins and modulate Kv4 channel trafficking, surface expression, and gating function (Nadal et al., 2003; Jerng et al., 2004b; Ren et al., 2005; Zagha et al., 2005). Importantly, DPLPs profoundly and fundamentally accelerate Kv4 channel activation and inactivation gating, as well as shifting the voltage dependence of activation and inactivation in the hyperpolarizing direction, accelerating inactivation, altering channel conductance, and changing toxin sensitivity (Kaulin et al., 2009; Maffie et al., 2013). Although DPLPs form homo- or heterodimers in solutions (Strop et al., 2004), the relationship between structures in solution and those fully integrated into the channel remains unknown. Nevertheless, Soh and Goldstein (2008) studied the functional properties of channels from Kv4 and DPP6 subunits linked in tandem as well as the molar ratios of DPP6 and Kv4.2 subunits purified from assembled channels and concluded that channel complexes are composed of four subunits of Kv4.2 and DPP6. Studies of heteromultimeric channel complexes with DPP6a and DPP6K also suggest that there are four DPP6 subunits per channel (Jerng and Pfaffinger, 2012).

**FIGURE 1:**
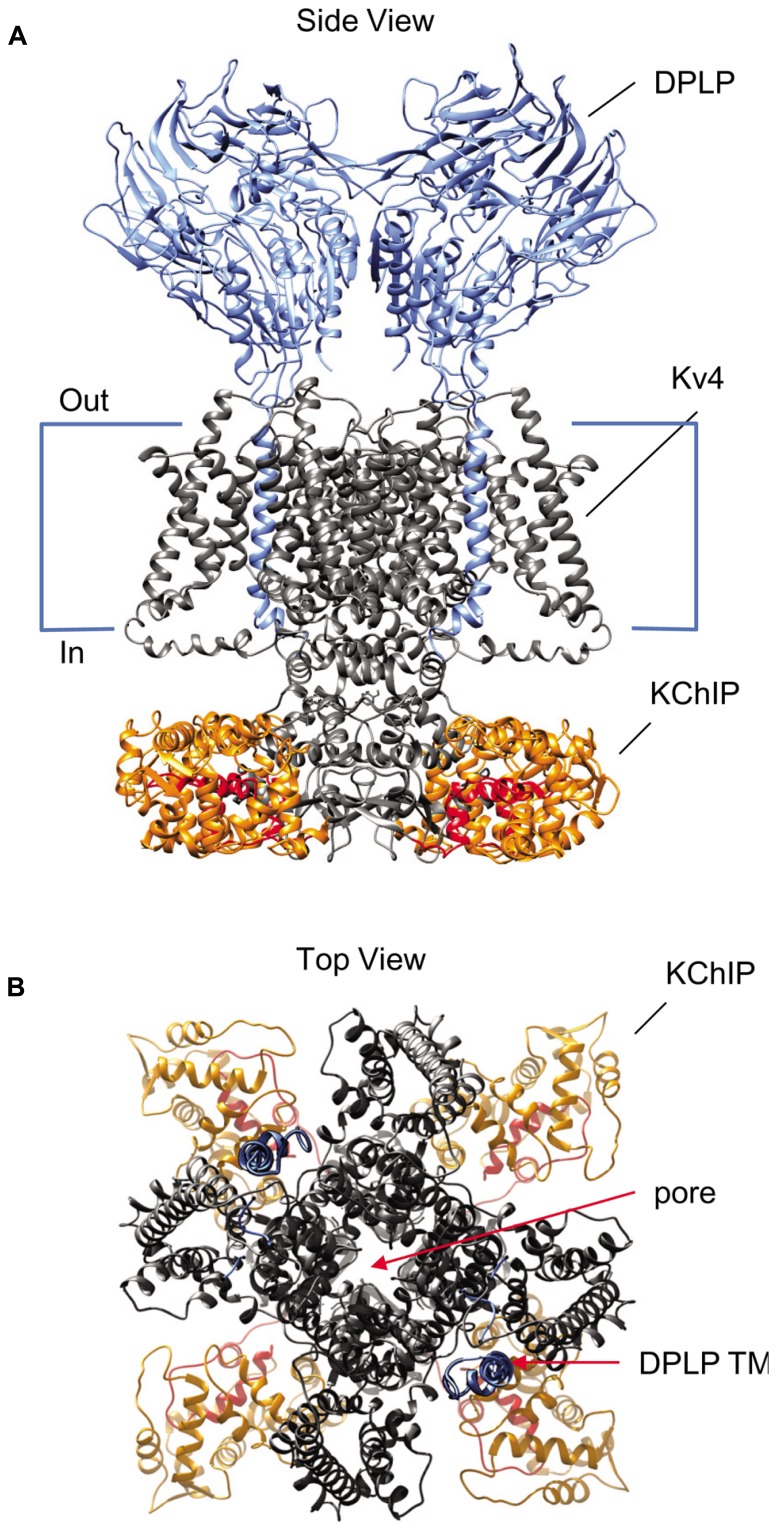
**Structural model of the Kv4-KChIP-DPLP supermolecular complex.** The structure of the KChIP1-Kv4.3 T1 complex from Pioletti et al. (2006) was docked with the Kv1.2 structure from Long et al. (2007). The structure of DPP6 dimer from Strop et al. (2004) is positioned so that the transmembrane segments are near the voltage-sensing domains of the channel core domains. The structure depicts only two DPP6 molecules, while the experimental evidence suggests that complete complex contains four DPLP subunits (Soh and Goldstein, 2008). **(A)** Side view of the multi-protein channel complex. Kv4 pore-forming subunit is shown in gray, with the exception of the pre-T1 N-terminal domain which is shown in red. The KChIP molecules are shown in orange; the DPLP molecules, blue. This view clearly shows that KChIP binding sequesters the Kv4 N-terminus. **(B)** Top view of the complex as seen from the extracellular side. The central pore generated by the Kv4 subunits is indicated and can be easily observed. Note the KChIP molecules (orange) and the DPLP transmembrane domains (TM, blue) reside between the Kv4 voltage sensors with free access to the T1 side windows.

Multiple lines of evidence suggest that the KChIP and DPLP auxiliary subunits work in concert to modulate Kv4 channel function, and that the Kv4, KChIP, and DPLP subunits together form the macromolecular complex responsible for producing I_SA_ (**Figure 1**). Kv4, KChIP, and DPLP transcripts and proteins have overlapping expression patterns throughout the CNS (Rhodes et al., 2004; Zagha et al., 2005; Clark et al., 2008). Kv4, KChIP, and DPLP proteins can be co-immunoprecipitated from brain samples (Nadal et al., 2003; Jerng et al., 2005; Marionneau et al., 2009). Co-assembly of Kv4, KChIP, and DPLP subunits can generate K^+^ currents in heterologous studies with functional properties similar to those found in native I_SA_ (Nadal et al., 2003; Jerng et al., 2005). In cultured cerebellar granule (CG) cells and hippocampal neurons, genetic knockout and RNAi of DPP6 simultaneously reduces the protein level of all ternary channel complex subunits (Nadin and Pfaffinger, 2010; Sun et al., 2011). Meanwhile, genetic knockout of Kv4 expression leads to downregulation of KChIP protein levels (Menegola and Trimmer, 2006; Norris et al., 2010).

In addition to KChIP and DPLP proteins, various published reports suggest that additional ancillary, cytoskeletal, and molecular chaperone proteins may interact with the I_SA_ channel complex, including Kvβ subunit, Navβ1 subunit, MinK-related protein 1 (MiRP1), kinesin isoform Kif17, Kv channel-associated protein (KChAP), post-synaptic density protein 95 (PSD-95), and filamin (Petrecca et al., 2000; Yang et al., 2001; Zhang et al., 2001; Deschenes and Tomaselli, 2002; Wong et al., 2002; Marionneau et al., 2012a; **Table 1**). All of these proteins have been reported to co-immunoprecipitate with Kv4 subunits from native tissues or transfected culture cells, suggesting that these candidates either directly interact with Kv4 proteins or associate indirectly as part of the large multi-protein complex. However, questions remain as to whether these proteins play a fundamental role in the function of I_SA_. Compared to KChIPs and DPLPs, their overall abundance in the I_SA_ complex in the brain appears to be low or undetectable (Marionneau et al., 2012b). In addition, the chaperone and cytoskeletal proteins tested so far (KChAP, PSD-95, Kif17, filamin) exert minimal effects on functional properties and increase peak current amplitude significantly less than KChIPs and DPLPs (**Table 1**). Furthermore, studies on the ancillary proteins Kvβ1, Navβ1, and MiRP1, well-known partners of Kv1, Nav, and KCNQ channels, respectively, have yielded conflicting results for their modulatory effects on Kv4 channels. Their facilitative effect on Kv4 peak current is highly variable, between 0.2- to 3-fold increase and significantly less than KChIPs and DPLPs. Moreover, unlike KChIPs and DPLPs, their molecular interaction with Kv4 channels remains controversial. For example, based on crystal structures, Kvβ1 interacts with the T1 domains of Kv1 channels (Gulbis et al., 2000); however, the motif used by Kv1 channels is not conserved in Kv4 channels, and it is currently unclear whether Kvβ interacts with Kv4 channels at the N- or C-terminal domain (Nakahira et al., 1996; Yang et al., 2001; Wang et al., 2003). Finally, it is possible that the regulatory effects observed under high expression conditions in heterologous cells are not normally seen in the nervous system. For these reasons, discussions of these other ancillary proteins are not included in this review.

**Table 1:** Validation of auxiliary subunits and accessory proteins.

Proteins	Kv4.2-IP PAF	Kv4 CoIP	Δ Ipeak	Δ Functional properties
**Auxiliary subunits**				
KChIP1	–	Brain	↑ 7.5-fold	Slowed inactivation; rightward shift in SSI; accelerated recovery; accelerated closing (An et al., 2000; Holmqvist et al., 2002; Morohashi et al., 2002)
KChIP2	1.7	Brain	↑ 5.5-fold	
KChIP3	1.4	Brain	↑ 5.8-fold	
KChIP4	5.6	Brain	↑ 2- to 3-fold	
DPP6	5.3	Brain	↑ 4- to 18-fold	Accelerated activation and inactivation; leftward shifts in SSI and G-V relationship; accelerated recovery; accelerated channel closing (Nadal et al., 2003,^2006^; Jerng et al., 2004b; Zagha et al., 2005)
DPP10	2.0	Brain	↑ 5.9-fold	
**Accessory proteins**				
Kvβ1	0.7	Brain	↑ 0.2- to 3-fold	Moderate acceleration of inactivation; moderate to no effect on SSI; no effect on recovery; Kv1 preference (Nakahira et al., 1996; Yang et al., 2001; Wang et al., 2003; Aimond et al., 2005)
Navβ1	0.8	Brain Heart	↑ 0.3- to 2-fold	Inconsistent effects reported (Chabala et al., 1993; Deschenes and Tomaselli, 2002; Deschenes et al., 2008; Hu et al., 2012; Marionneau et al., 2012a)
MiRP1	–	*In vitro*	Variable	Inconsistent effects reported (Zhang et al., 2001; Deschenes and Tomaselli, 2002; Radicke et al., 2006)
KChAP	–	Heart	↑ 0.8-fold	None (Kuryshev et al., 2000,^2001^)
NCS-1	–	Heart	↑ 0.5-fold	Inconsistent effects reported (Nakamura et al., 2001; Guo et al., 2002; Ren et al., 2003)
PSD-95	–	*In vitro*	↑ 2-fold	None (Wong et al., 2002; Wong and Schlichter, 2004)
Filamin	–	Brain	↑ 2.5-fold	None (Petrecca et al., 2000)
Kinesin isoform Kif17	–	Brain	ND	ND (Chu et al., 2006)

*PAF, protein abundance factor; PAF (Kv4.2) = 2.1 (Marionneau et al., 2011); (–), not detected; ND, not determined; SSI, steady-state inactivation; G-V, conductance-voltage.*

*Other proteins (Gabra-6, Gpr158, Prkcb1) have not undergone validation.*

## N-TERMINAL VARIANTS OF AUXILIARY SUBUNITS AND THEIR DIFFERENTIAL EXPRESSION PATTERNS

**FIGURE 2:**
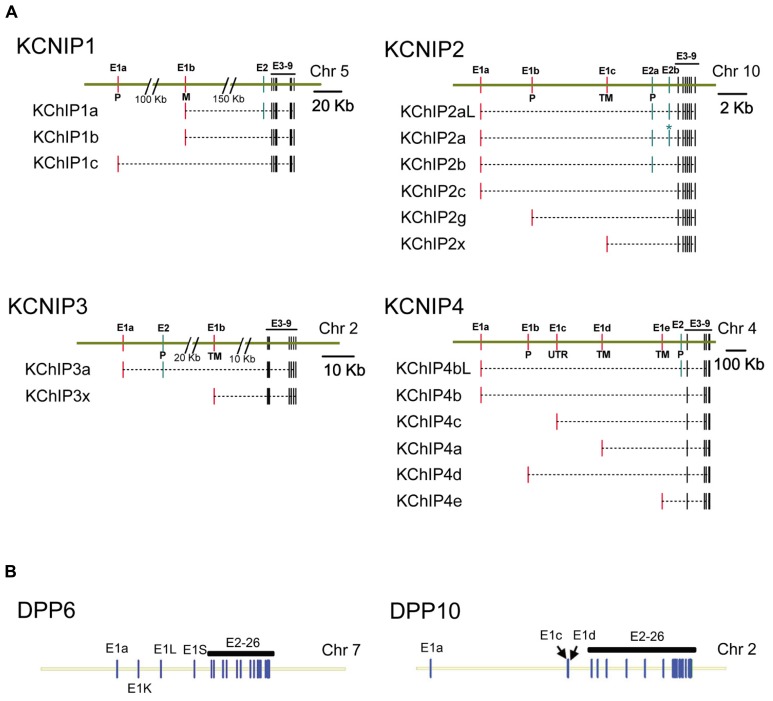
**Genomic structure of KChIP and DPLP genes.** Gene structures for Kv4.2 auxiliary subunit proteins show a common core with variable N-terminal exons. **(A)** KChIP genes show a common set of 3′ exons 3–9 with 1 to 2 alternative 5′ exons used to construct the final mRNA transcript. **(B)** DPLP genes show a common set of 3′ exons 2–26 with alternative 5′ first exons. Maps were constructed using exon locations in human chromosomes as given in Concensus CDS data base (http://www.ncbi.nlm.nih.gov/CCDS) or determined from published mRNA sequences using Splign (http://www.ncbi.nlm.nih.gov/sutils/splign). Additional splice variants of these genes have been proposed that are not shown. **(A)** KChIP1: KChIP1a-CCDS34286, KChIP1b-CCDS4374, KChIP1c-CCDS34285. KChIP2: KChIP2al-CCDS7521, KChIP2a-CCDS7522, KChIP2b-CCDS41562, KChIP2c-CCDS7524, KChIP2g-CCDS7525, KChIP2x- Jerng and Pfaffinger (2008). KChIP3: KChIP3a-CCDS2013, KChIP3x-CCDS33245. KChIP4: KChIP4bL-CCDS43216, KChIP4b-CCDS43215, KChIP4c-CCDS47035, KChIP4a-CCDS3428, KChIP4d-CCDS43217, KChIP4e- Jerng and Pfaffinger (2008). **(B)** DPP6: splicing based on Jerng et al. (2009). DPP10: DPP10a-CCDS46400, DPP10c-CCDS54388, DPP10d-CCDS33278. P, S-palmitoylation; M, N-myristoylation; TM, transmembrane.

Following their discovery, it was quickly realized that KChIP and DPLP proteins exist as N-terminal variants, products of different start sites and/or alternative splicing (Jerng et al., 2004a; Pruunsild and Timmusk, 2005; Pongs and Schwarz, 2010). All genes encoding KChIP and DPLP contain a core set of exons that are included in every transcript, but they also contain alternative promoters and optional exons that can be alternatively spliced into the final transcript (**Figure 2**). As a result, the different transcripts encode a set of proteins with variable N-termini attached to a common C-terminal core. As shown by Northern hybridization, RT-PCR, and *in situ* hybridization, the expression of N-terminal variant transcripts is not uniform throughout the brain; instead, each variant exhibits a specific expression pattern with variable levels of transcripts in different neuronal populations. Importantly, when combined with variant-specific functional effects (discussed later), the overlapping of distinct KChIP and DPLP variants is an important contributor to functional or regulatory differences in I_SA_ of different neurons.

### KChIPs

As members of the neuronal calcium sensor (NCS) gene family, KChIPs have existed as a distinct gene family predating the divergence of cnidarians such as Nematostella (An et al., 2000). In common with other members of the NCS-1 gene family, KChIP proteins have N- and C-terminal lobules each containing two EF-hands surrounding a deep hydrophobic pocket. Under normal circumstances, it appears that only EF-3 and EF-4 of the C-terminal lobule binds Ca^2^^+^; in the N-terminal lobule, EF-1 is unbound and EF-2 binds Mg^2^^+^. In mammals, four separate genes (KCNIP1, KCNIP2, KCNIP3, KCNIP4) encode the four families of KChIP proteins: KChIP1, KChIP2, KChIP3, and KChIP4 (**Figure 2A**). As for gene structure, all KChIP1–4 genes contain a homologs set of 7 C-terminal exons encoding the four EF-hands of the core domain, which are preceded by multiple transcription start sites and alternative exons (**Figure 2A**). The resulting transcripts create sets of 17 different proteins with variable N-terminal domains that comprise the most diverse class of Kv channel auxiliary subunits.

**FIGURE 3:**
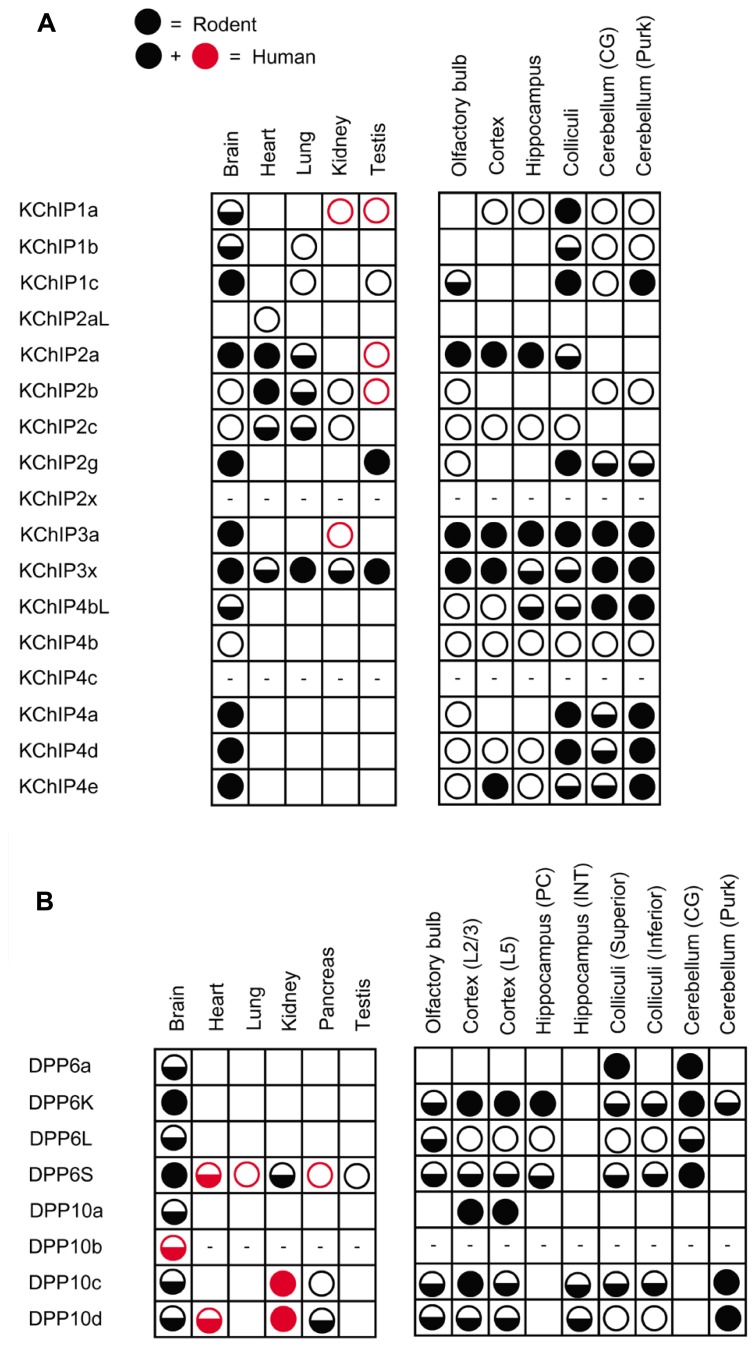
**Differential expression of auxiliary subunit N-terminal variants in various tissues and brain regions in mouse and human. (A)** Summary of the expression patterns of KChIP N-terminal variants in mouse (black) and human (black and red): KChIP1 (Van Hoorick et al., 2003; Pruunsild and Timmusk, 2005), KChIP2 (Takimoto et al., 2002; Pruunsild and Timmusk, 2005), KChIP3 (Pruunsild and Timmusk, 2005), and KChIP4 (Holmqvist et al., 2002; Pruunsild and Timmusk, 2005). **(B)** Summary of the expression patterns of DPLP N-terminal variants in mouse (black) and human (black and red): DPP6 (Wada et al., 1992; de Lecea et al., 1994; Kin et al., 2001; Radicke et al., 2005; Nadal et al., 2006; Maffie et al., 2009; Jerng and Pfaffinger, 2012) and DPP10 (Chen et al., 2006a; Takimoto et al., 2006; Jerng et al., 2007). No symbols, no expression; open circle, low expression; half-filled circle, moderate expression; filled circle, high expression; dash, not determined; CG, cerebellar granule cells; Purk, cerebellar Purkinje cells; PC, pyramidal neuron; INT, interneuron; L2/3, layer 2 and 3; L5, layer 5.

Further analysis of KChIP transcript expression in various tissues yields distinctive tissue specificity and expression patterns, as summarized in **Figure 3A** with references provided in the figure legend. Among the four KChIP families, only KChIP4 is expressed exclusively in the brain, while the others can be found in other tissues such as heart, lung, and testis. Although the mRNA transcripts of nearly all the KChIP N-terminal variants are detected in the brain, the expression levels vary significantly between neuronal populations, with some expressing strongly only in very specific neuronal populations. In addition, some KChIP variants exhibit preferential expression along the anterior–posterior axis. For example, whereas KChIP3a and KChIP3x transcripts have mostly strong expression throughout the brain regions, other variants show general preferences for expression in the forebrain (KChIP2a) or hindbrain (KChIP2g, KChIP4bL, KChIP4a, KChIP4d, KChIP4e).

**FIGURE 4:**
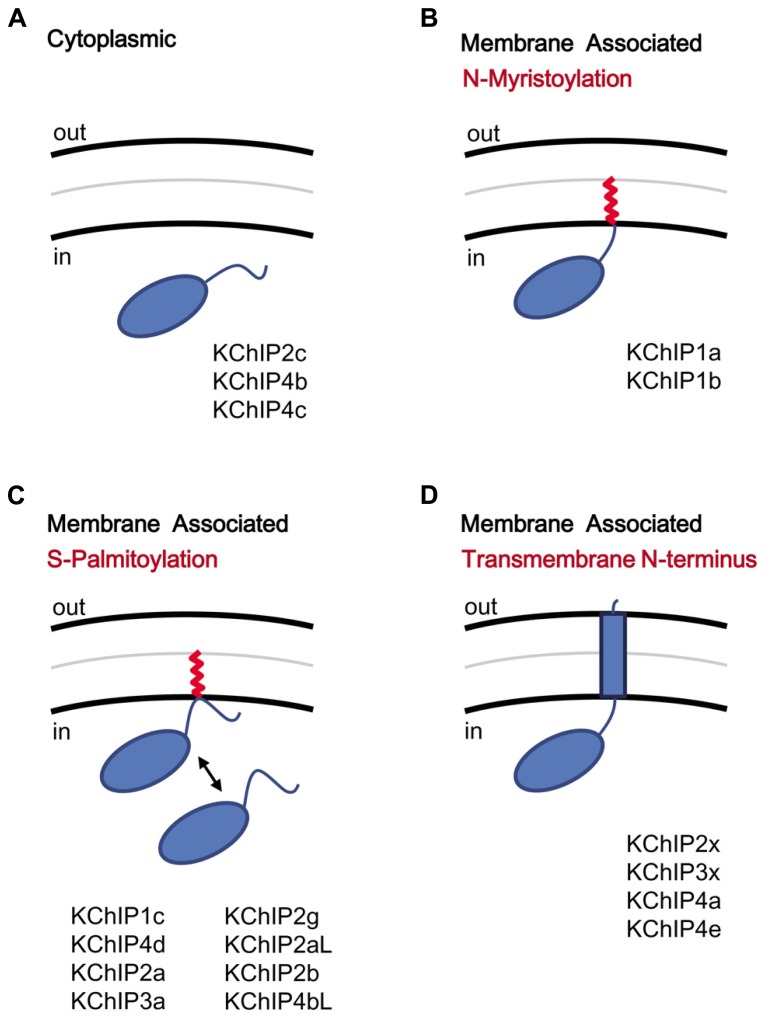
**KChIP N-terminal variants and the motifs coding for membrane association.** KChIPs can be freely cytoplasmic or membrane associated. **(A)** Cytoplasmic KChIPs lack the sequence motif to allowing anchoring of the KChIP molecule to the membrane. **(B)** Membrane association by N-myristoylation. The myristoylate group (red) is attached to a glycine residue at position 2. **(C)** Membrane association by S-palmitoylation. The palmitoylate group (red) is attached to a reactive cysteine within the N-terminal domain via a reversible reaction and induces membrane association. **(D)** Membrane association by insertion of a transmembrane N-terminal segment. Multiple N-terminal variants possess a highly hydrophobic segment that traverses the membrane. Aside from functioning as a membrane anchor, the transmembrane segment also modulates surface trafficking and channel gating.

Although the KChIP core is cytoplasmic by nature, the variable N-termini of KChIPs play a major role in controlling the protein’s subcellular localization by encoding motifs that induce the protein to associate with membranes (**Figure 4**). Mammalian KChIPs can be divided into four classes based on the presence or absence of specific N-terminal membrane association motifs: cytoplasmic, N-myristoylated, S-palmitoylated, and transmembrane. N-myristoylation occurs in KChIP1a and KChIP1b when a shared glycine residue at position two is modified with a myristoylate group (O’Callaghan et al., 2003; **Figure 4B**). N-terminal myristoylation is a common feature of other NCS-1 family proteins and is often seen in invertebrate KChIPs; however, only these two KChIP1 variants are known to be myristoylated in mammals. In other NCS-1 family proteins, like recoverin, the membrane association can be regulated by a Ca^2^^+^-myristoyl switch, whereby Ca^2^^+^ binding to EF-hands 2 and 3 leads to sequestration of the fatty acid chain by the protein’s hydrophobic pocket (Tanaka et al., 1995). However, KChIP1a and KChIP1b appear to remain membrane-anchored regardless of the Ca^2^^+^ concentration, and N-myristoylation causes the KChIP1 proteins to localize to the post-ER trafficking vesicles, possibly the secretory vesticles of the Golgi body (O’Callaghan et al., 2003). It is hypothesized that N-myristoylation of these KChIPs help regulate the trafficking of Kv4 channels to the plasma membrane.

A second form of fatty acid modification of KChIPs, S-palmitoylation, appears to have evolved more recently but prior to the divergence of vertebrate KChIPs into different families (**Figure 4C**). S-palmitoylation motifs are found in both Exon 1 and Exon 2 of different KChIP genes, based on analysis by a palmitoylation site prediction algorithm (CSS-Palm 4.0) and mutational analysis (Takimoto et al., 2002; Ren et al., 2008). S-palmitoylation is a major post-translational modification (8 out of 17 variants) of the KChIP N-terminal variants, and for S-palmitoylated variants, the fatty acid attachment is required for the facilitation of Kv4 expression at the cell surface by KChIPs (Takimoto et al., 2002). Interestingly, the S-palmitoylation of these KChIPs may have important implications in the trafficking of Kv4 channel complexes to post-synaptic lipid rafts in the brain. Kv4.2 from rat brain preparations is found in the lipid rafts, a detergent-insoluble glycolipid-enriched membrane fraction (Wong and Schlichter, 2004), but the recruitment of Kv4.2 to lipid rafts cannot be explained by an interaction between Kv4.2 and the major post-synaptic density protein (PSD-95). Since S-palmitoylated proteins are known to accumulate in the lipid rafts, perhaps S-palmitoylation of KChIPs play an important role in transporting Kv4 channels to lipid rafts. Furthermore, S-palmitoylation is a reversible process, suggesting that trafficking of Kv4 channels may be regulated by the cell. Indeed, studies by Pruunsild and Timmusk (2012) using over-expression of tagged KChIP variants in neurons indicate that, while N-myristoylated and transmembrane KChIPs consistently show respectively cytoplasmic punctuate and perinuclear localization, many of the supposed S-palmitoylated KChIPs were diffusedly distributed throughout the neuron (Pruunsild and Timmusk, 2012).

In addition to N-myristoylation and S-palmitoylation, some KChIP N-terminal variants (KChIP2x, KChIP3x, KChIP4a, KChIP4e) contain a transmembrane domain capable of modulating both surface expression and gating (**Figure 4D**). Based on sequence similarity, it is likely that the transmembrane-encoding N-terminal exons of KChIP2x, KChIP3x, and KChIP4a have been retained from the ancestral KChIP gene, and they all show similar functional effects, including ER retention, suppression of Kv4 functional expression, and modulation of channel gating properties (Jerng and Pfaffinger, 2008). KChIP4e, which is also predicted to have a transmembrane domain, likewise has a perinuclear subcellular localization, even though a fusion protein between the KChIP4e variable N-terminus with eGFP was not found to associate with the membrane fraction (Pruunsild and Timmusk, 2012). In summary, while N-myristoylation and S-palmitoylation can specifically promote trafficking of KChIPs to the post-ER or the Golgi bodies, the transmembrane segment found in KChIP2x, KChIP3x, and KChIP4a affect both channel gating as well as trafficking.

### DPLPs

The two DPLP genes, DPP6 and DPP10, are homologous genes with significant sequence identity and are likely products of gene duplication early during the evolution of vertebrates (**Figure 2B**). The overall genomic structures of DPP6 and DPP10 are strikingly similar, with alternative first Exons encoding the variable cytoplasmic N-termini and a common core set of 25 exons encoding the transmembrane domain and globular extracellular domain (Takimoto et al., 2006; Jerng et al., 2009). Exon 1a shares significant functional and sequence homology between DPP6 and DPP10 and thus likely predates the ancestral split into separate genes (Jerng et al., 2009). Thus far, five DPP6 (DPP6a, DPP6K, DPP6L, DPP6D, DPP6S) and four DPP10 (DPP10a, DPP10b, DPP10c, DPP10d) N-terminal variants have been isolated in mammals for a combined total of nine variants, and all but DPP6D has been functionally characterized (Nadal et al., 2006; Takimoto et al., 2006).

The expression profiles of DPP6 and DPP10 N-terminal variants have revealed some interesting findings (**Figure 3B**). First, the precise DPLP N-terminal variants expressed vary even between mammalian species. For example, DPP10c is found in human but not in rodents (Takimoto et al., 2006). Second, in both humans and mice, DPP6S, DPP10c, and DPP10d are widely expressed in non-brain tissues (Wada et al., 1992; Radicke et al., 2005; Takimoto et al., 2006). Interestingly, the first exons that encode DPP6S and DPP10d N-termini are the alternative exons closest to Exon 2, suggesting that perhaps they share a promoter with decreased specificity for brain expression. Third, the expression patterns of DPP6a and DPP10a paralogs are the most specific; DPP10a is expressed specifically in the cortex, whereas DPP6a is expressed in superior colliculus, CG cells, red nucleus, and other specific brain structures (Jerng et al., 2007; Maffie et al., 2009; Jerng and Pfaffinger, 2012). Fourth, DPP6 prefers to be expressed in neurons that also express Kv4.2, such as hippocampal pyramidal neurons and CG cells (Nadal et al., 2006; Clark et al., 2008). DPP10 prefers to be expressed in neurons that also express Kv4.3, such as hippocampal interneurons and cerebellar Purkinje neurons (Zagha et al., 2005).

## HOW Kv4 FUNCTIONAL PROPERTIES ARE MODULATED BY AUXILIARY SUBUNITS CORE REGIONS

In the past two decades, tremendous progress has been made in our understanding of the molecular basis underlying the functional properties of Kv channels. Kv4 channels, like other voltage-gated K^+^ channels, are rotationally symmetrical homo- or heterotetrameric arrangements of Kv4.1, Kv4.2, and Kv4.3 pore-forming α-subunits (Long et al., 2007; **Figure 1**). The Kv4 pore-forming subunits consist of cytoplasmic N- and C-terminal domains and a central transmembrane core region consisting of six transmembrane segments (S1–S6), a pore loop region (P), and the associated inter-segment linkers. The transmembrane core region can be divided into the voltage-sensing region (S1–S4) and the pore-lining region (S5-P-S6; Kubo et al., 1993). The pore-lining region of Kv channel α-subunits contains the minimal elements required for K^+^ selectivity, conductance, and certain forms of inactivation. In the cytoplasmic N-terminus, the T1 domain controls the organized subfamily specific tetramerization of Kv4 subunits and plays an important role in KChIP protein assembly (Shen and Pfaffinger, 1995). In the mature channel, the T1 domain hangs down from the central axis of the channel core in a manner that has been likened to a gondola. The structural organization of the cytoplasmic C-terminal portion of the channel is less well understood, but this region contains important phosphorylation sites as well as a PDZ-binding motif (Jerng et al., 2004a).

Structure-function studies in heterologous expression systems have contributed significantly to our understanding of how KChIP and DPLP auxiliary subunits modulate the Kv4 channel. Generally, these studies have shown that auxiliary subunits have certain effects that are attributed to the core domain and thus common to all variants of a particular subunit class, as well as additional modulatory effects that are only produced by certain N-terminal variants. We will first consider the effects that are produced in common.

### MODULATION OF EXPRESSION AND GATING BY THE KChIP CORE DOMAIN

When Kv4 channels are expressed alone in heterologous systems, the N-terminal portion of the peptide forms an intracellular pore blocker that produces open-state inactivation (OSI) similar to the rapid “ball-and-chain” N-type inactivation of Shaker channels (Gebauer et al., 2004). In addition to producing N-type inactivation, the N-terminal domain also performs a separate function, one of suppressing the surface expression of Kv4 channels by inducing ER retention (Shibata et al., 2003). Both of these N-terminal functions are suppressed by KChIP molecules. KChIP proteins use their hydrophobic pockets to bind the hydrophobic Kv4 N-terminal domain, with critical interactions involving highly conserved Trp8 and Phe11 residues in the Kv4 channel N-terminus (**Figure 1**; Scannevin et al., 2004; Zhou et al., 2004; Pioletti et al., 2006; Wang et al., 2007). When all four Kv4 N-termini are bound to KChIPs, the channel complex is released from ER retention and placed at high levels on the cell surface (Kunjilwar et al., 2013). As expected, these channels also have slowed inactivation due to the sequestration of the Kv4 N-terminus.

In addition, KChIP binding accelerates recovery from inactivation by modulating a second inactivation phenomenon present in Kv4 channels often described as closed-state inactivation (CSI). As opposed to OSI, CSI occurs while the channel is undergoing activation or deactivation transitions, and it has been proposed to involve a decoupling of voltage sensor movement from channel opening (Jerng et al., 1999; Bahring et al., 2001; Bahring and Covarrubias, 2011). Channels that undergo N-type inactivation often transition over to CSI, and thus recovery from this state is a key determinant of inactivation recovery kinetics. While the exact mechanism of KChIP-mediated acceleration of recovery is not entirely clear, a key factor appears to involve KChIP interactions at a second binding site on the side of the Kv4 subunit T1 domain (Site 2; Scannevin et al., 2004; Pioletti et al., 2006; Wang et al., 2007). KChIP protein binding at Site 2 bridges two T1 domains, stabilizing the T1 domain assembly interface and helps to drive Kv4 channel assembly. The T1 domain has been shown to affect recovery from CSI, and it is most likely that the KChIP-mediated stabilization of the T1 domain structure restricts its potential conformations and leads to an acceleration of recovery kinetics.

### MODULATION OF EXPRESSION AND GATING BY THE DPLP CORE DOMAIN

Using their single-pass transmembrane domains, DPLPs interact with Kv4 channels via the channel core transmembrane region rather than the cytoplasmic N-terminus used by KChIPs (**Figure 1**; Dougherty et al., 2009). As for the sites of interaction on Kv4 subunits, some preliminary evidence suggests that the DPLP transmembrane domain is interacting with portions of the S1 and S2 segments, although the exact binding site is not yet clear (Ren et al., 2003). Since DPLPs can affect both single-channel conductance and toxin binding, two properties that depend on the pore structure, it is likely that DPLP transmembrane binding has an impact on the packing of the transmembrane pore domain. These packing effects may restrict the intermediate gating states available to the channel, thereby accelerating the channel gating kinetics.

The interaction between DPLP and Kv4 subunits also promote surface expression of Kv4 channels in heterologous expression studies, similar to the effects of KChIP co-expression; however, the mechanism appears to be different and does not involve sequestration of the Kv4 channel N-terminus (Kunjilwar et al., 2013). Instead, it appears that DPLP-mediated enhancement of Kv4 expression depends on the glycosylation state of the channel. By binding to the Kv4 channel, DPLPs provide a large extracellular domain with multiple glycosylation sites and, in effect, alter the glycosylation state of the channel. The trafficking effects of DPLPs, at least in part, depends on proper glycosylation of the DPLP extracellular domain, since treatment with tunicamycin or mutations that disrupt glycosylation results in a loss of the enhanced expression associated with DPLPs (Cotella et al., 2010; Kunjilwar et al., 2013).

## FUNCTIONAL IMPACT OF THE N-TERMINAL “TOOL KIT” AND THEIR MECHANISM OF ACTION

Kv channel-interacting protein and DPLP N-terminal variants have distinct expression patterns and unique functional effects that are likely important for shaping channel functional properties, post-translational regulation, and subcellular localization. Some of the unique properties of KChIP and DPLP variants have been identified in heterologous expression systems, but other functional effects may require native expression environments to be observed. Since most of the variable N-terminal exons found in KChIP and DPLP genes are highly conserved throughout vertebrate evolution, it is very likely that every variant encodes some important specialized functions. In this section, we will discuss some of the known functional effects of specific N-terminal variants.

### KChIP2x, KChIP3x, KChIP4a, AND KChIP4e: THE tmKChIPs

**FIGURE 5:**
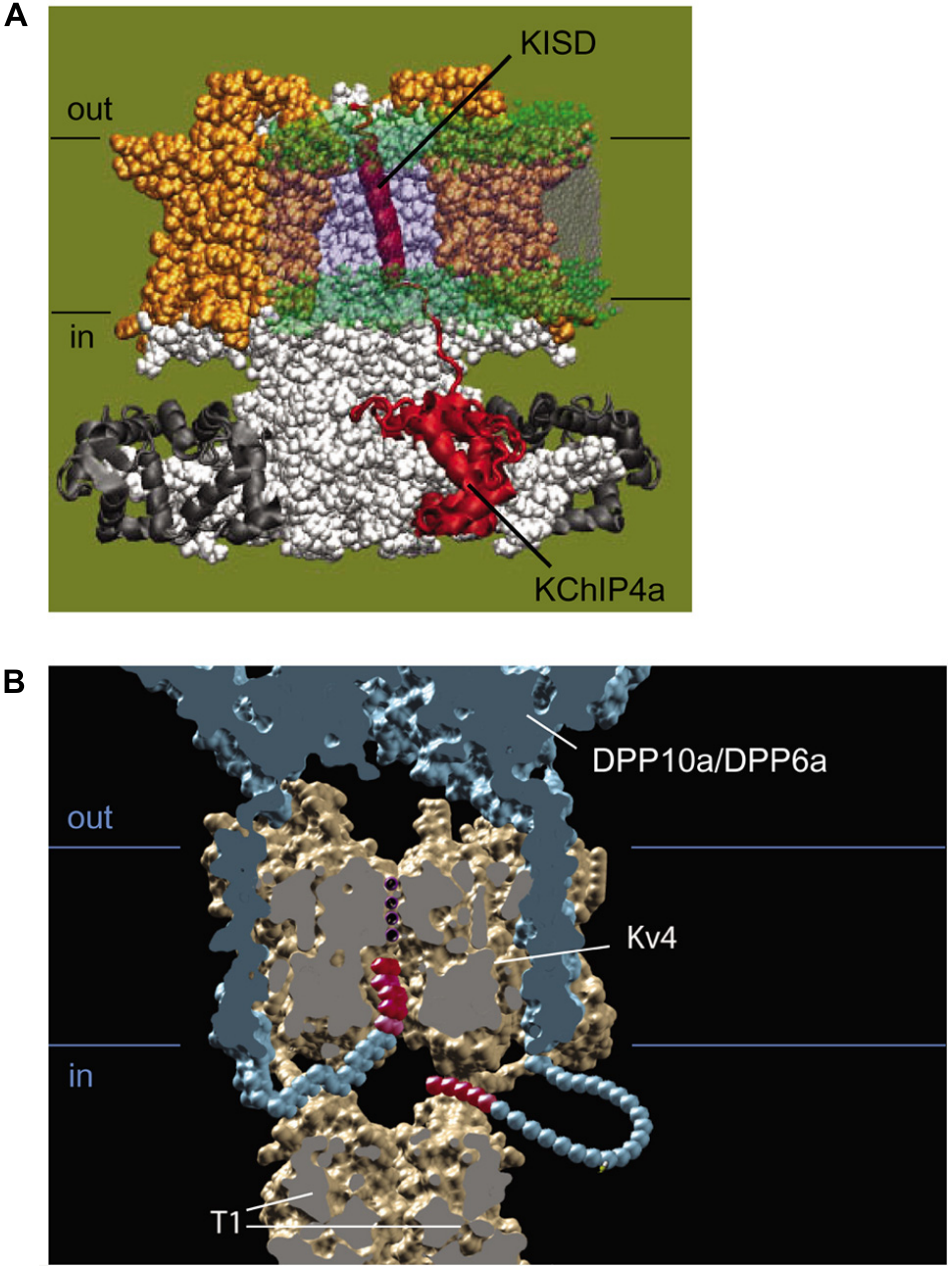
**Molecular mechanism of functional regulation by KChIP4a, DPP6a, and DPP10a. (A)** Working molecular model of the transmembrane Kv-channel inactivation suppressor domain (KISD) inserting through the membrane and making contacts with the Kv4 channel core, as adapted from Jerng and Pfaffinger (2008). As reported by Tang et al. (2013), separate domains within the KISD determine ER retention and regulation of channel gating. KChIP4a is shown in red, voltage sensors in yellow, and the remaining Kv4 channels in white. **(B)** Side view of a model illustrating the basis for fast inactivation mediated by DPP6a and DPP10a. Two of the four subunits have been removed to reveal the inner channel structure, including the permeation pathway with K^+^, DPLP extracellular and transmembrane domain (blue), and the DPLP N-termini with inactivation particles (red).

The variable N-terminal domains of KChIP2x, KChIP3x, and KChIP4a feature a stretch of hydrophobic amino acids that folds into a transmembrane segment, facilitates membrane partition, and promotes ER retention (**Figure 5A**; Jerng and Pfaffinger, 2008; Pruunsild and Timmusk, 2012). In intact cells, biotinylation studies of N-terminal cysteine mutants show that the KChIP4a N-terminus is extracellular and the transmembrane segment begins with leucine at residue 3. In their *in vitro* studies of KChIP4a, Schwenk et al. (2008) and Liang et al. (2009) show that a 21-residue-long segment beginning with Leu-3 or Glu-4 forms a 6-turn α-helix in solution NMR and in X-ray crystallography, and under *in vitro* conditions, this helix can fold back onto the core domain and bind the same hydrophobic pocket that sequesters Kv4 N-terminal domain (Schwenk et al., 2008; Liang et al., 2009). However, this autoinhibitory state of KChIP4a appears short-lived, since the Kv4.3 N-terminus can out-compete the KChIP4a N-terminus for the hydrophobic pocket and free the KChIP4a N-terminus (Liang et al., 2009), thereby allowing it to become ultimately anchored in the membrane. The final N-terminal variant with a putative transmembrane N-terminus, KChIP4e, is considerably different from the paralogs of KChIP2x, KChIP3x, and KChIP4a. Based on analysis by The Eukaryotic Linear Motif website (http://elm.eu.org), the putative transmembrane domain of KChIP4e begins at residue 10 and ends at 32, giving a longer extracellular N-terminal stretch. Because the transmembrane nature of their N-termini, in this review these variants are collectively referred to as transmembrane KChIPs, or tmKChIPs.

Although KChIP2x, KChIP3x, and KChIP4a are in many ways functionally similar, specific functional differences between these tmKChIPs and KChIP4e suggest that the regulatory effects involve more than just the insertion of transmembrane domains into the lipid bilayer. For example, even though KChIP4e has a putative transmembrane N-terminus and shows perinuclear localization, KChIP4e increases surface expression of Kv4.2 channels, does not slow inactivation kinetics, and accelerates recovery from inactivation (Jerng and Pfaffinger, 2008; Pruunsild and Timmusk, 2012). Also, while KChIP2x and Kv3x slow inactivation similar to KChIP4a, they accelerated recovery like typical KChIPs (Jerng and Pfaffinger, 2008). It therefore seems likely that certain differences in the N-terminal sequence may confer different properties to the channel complex, perhaps by altering how the transmembrane region of the protein interacts with the transmembrane portion of the channel or other membrane regulatory proteins. The difference in surface expression between KChIP4e and the other tmKChIPs may be due to differences in specific ER retention motifs between these variants (Shibata et al., 2003; Jerng and Pfaffinger, 2008; Liang et al., 2010; Pruunsild and Timmusk, 2012; Tang et al., 2013). Indeed, Tang et al. (2013) have identified the ER retention motif which consists of six hydrophobic and aliphatic residues (LIVIVL) within KChIP4a N-terminus (Tang et al., 2013). These residues are also highly conserved within KChIP2x and KChIP3x but not in KChIP4e.

Differences in functional effects may also reflect specific regulatory sequences. Residues 19–21 of KChIP4a (VKL motif) have been identified as those responsible for promoting CSI of Kv4.3 channels, with Leu-21 being the critical residue (Tang et al., 2013). CSI is the predominant form of inactivation for Kv4 channels bound to KChIPs (Kaulin et al., 2008). The similarity in the sequence between KChIP4a (VKL) and KChIP2x (VKV), and KChIP3x (IAV) may explain why all these variants slow the inactivation time courses whereas the more distinct sequences in KChIP4e (LLH) at the homologous position does not. Lys-20 is conserved between KChIP4a and KChIP2x and may explain why the inactivation time courses are more similar between Kv4.2+KChIP4a and Kv4.2+KChIP2x than Kv4.2+KChIP3x (Jerng and Pfaffinger, 2008). Also, Val-21 for both KChIP2x and KChIP3x may explain why their steady-state inactivation (SSI) properties are vastly different from that of KChIP4a. In summary, available evidence suggests that discrete portions of the tmKChIP transmembrane domain induces ER retention and interacts with Kv4 pore-forming subunits to produce dramatic changes in the functional properties. At present, despite the widespread expression of tmKChIPs throughout the brain and the potential impact they have on I_SA_ channel expression and gating in neurons, nothing is known about the binding partner for the tmKChIP transmembrane domain on the Kv4 subunits, and further research is required to elucidate the precise interactions needed for tmKChIP-mediated modulation.

### DPP10a AND DPP6a: DPLPs MEDIATING N-TYPE INACTIVATION

**FIGURE 6:**
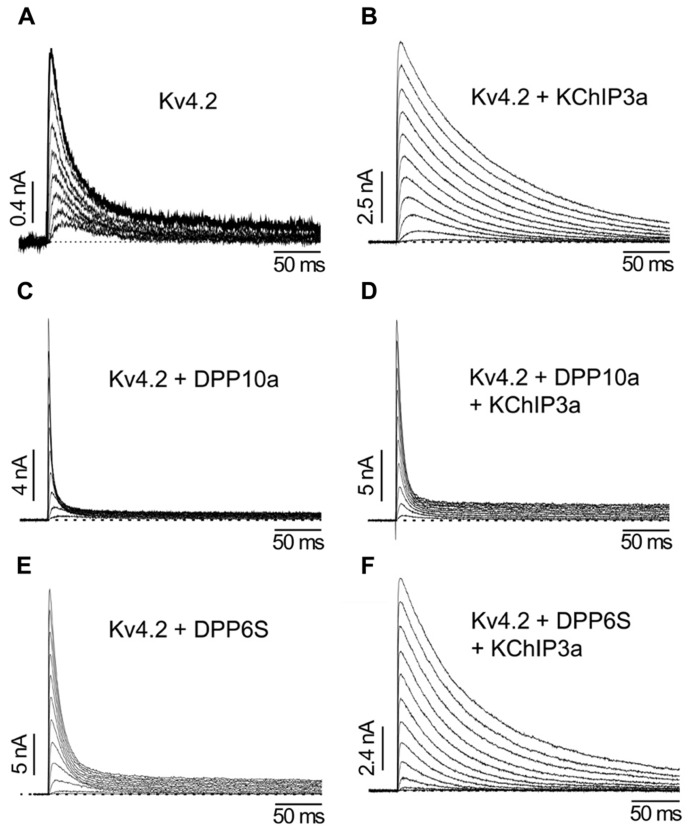
**The differential functional effects of DPLP N-terminal variants (Adapted from Jerng et al., 2005). (A)** Kv4.2 outward currents elicited by membrane depolarization in CHO cells. **(B)** After co-expression with KChIP3a, Kv4.2 channels significantly increase in surface expression and exhibit slower inactivation. **(C)** Similar to KChIP3a, co-expression of Kv4.2 with DPP10a results in a dramatic increase in peak current; however, the inactivation kinetics is markedly accelerated. **(D)** Channel complexes co-expressing KChIP3a and DPP10a results in currents that are more like that of Kv4.2+DPP10a channels, indicating the dominance of DPP10a-mediated fast inactivation. **(E)** DPP6-S accelerates inactivation of Kv4.2 channels. **(F)** Co-expression of DPP6-S with Kv4.2+KChIP3a channels does not produce dramatic acceleration of inactivation observed with DPP10a.

Although the transmembrane domain of DPLP imparts most of the functional effects associated with DPLP co-expression, such as accelerated gating kinetics and hyperpolarizing shifts in the voltage-dependence of activation and inactivation, early experiments studying DPP10a found significantly faster inactivation kinetics than DPP6S (Jerng et al., 2004b). This fast inactivation associated with DPP10a is transferrable to DPP6S by transplanting the DPP10a N-terminus, and among the DPP10 N-terminal variants, only DPP10a produced this effect (Jerng et al., 2004b, 2007). The DPP6a variant also confers a similar fast inactivation, suggesting that a conserved determinant within the N-terminus is responsible (Jerng et al., 2009; Maffie et al., 2009). This N-terminus-mediated fast inactivation occurs when Kv4 is bound by KChIP or in the absence of the endogenous Kv4 N-type inactivation, suggesting a direct action of the N-terminus (**Figure 6**; Jerng et al., 2009). This direction action by the N-terminus is a dominant feature in heteromeric DPLP channels, suggesting that fast inactivation can be generated with even less than 4 DPP10a or DPP6a on a single channel (Jerng et al., 2007; Jerng and Pfaffinger, 2012). Finally, deletion of the N-terminal 5 residues is sufficient to prevent the DPP10a- or DPP6a-mediated fast inactivation, and perfusion of the N-terminal peptide can also produce block (Jerng et al., 2009).

How do DPP10a and DPP6a mediate fast inactivation? Fast inactivation of K^+^ channels is commonly produced by a “ball-and-chain” or N-type mechanism, whereby the distal N-terminus occludes the inner pore and produce inactivation (Hoshi et al., 1990). Furthermore, in Shaker K^+^ channels, it has been shown that an internal blocker called tetraethylammonium (TEA) can compete with the blocking particle and the degree of block is proportional to the slowing of inactivation (Demo and Yellen, 1991). Moreover, the blocking particle is trapped by the channel closing during repolarization, and the relief from block is equivalent to channel recovery as observed in the tail current. Diagnostic tests prove that DPP10a and DPP6a confer fast inactivation by this Shaker N-type inactivation mechanism (Jerng et al., 2009); therefore, fast inactivation mediated by DPP6a or DPP10a is produced when the N-terminal inactivation particle blocks the open pore (**Figure 5B**). In addition, heterologous expression studies show that, similar to Shaker N-type inactivation found in Kv1.4, DPP6a- and DPP10a-conferred N-type inactivation is regulated by oxidation and reduction through a highly conserved N-terminal cysteine at position 13 (Ruppersberg et al., 1991; Jerng et al., 2012). These results suggest that (1) neurons expressing these DPLP variants are likely to have I_SA_ with fast inactivation, and (2) this fast inactivation is sensitive to the cell’s redox state.

### DPP6K

DPP6K modifies the functional properties of Kv4 channels in a very distinct way. Co-expressed with Kv4.2 channels, DPP6K produces the typical modulatory effects of increasing the current amplitude, decreasing the time to peak current, accelerating inactivation kinetics, and introducing leftward shifts in SSI and conductance-voltage relationship (Nadal et al., 2006). However, when compared to DPP6S and DPP6L, the recovery kinetics in the presence of DPP6K is significantly slower and the voltage dependence of SSI is markedly more hyperpolarized. In the Kv4-KChIP3a-DPP6K ternary complex, the unique DPP6K effects are even more pronounced (Jerng and Pfaffinger, 2012). Unlike other DPP6 variants, DPP6K dampens the ability of KChIP3a to promote an acceleration of recovery from inactivation and shift SSI to more positive potentials. Named after a high content of lysines in the N-terminus, DPP6K has four lysine residues (Lys-2, Lys-4, Lys-8, and Lys-11) within the 17-residue-long variable N-terminus (Nadal et al., 2006). However, alanine substitutions of these lysines have no effect on their modulation of Kv4 channel complexes, and the significance of these lysine residues remains certain (Jerng and Pfaffinger, 2011). Instead, deletion and point mutation analysis shows that the DPP6K functional effects come from a stretch of residues between Met-12 and Met-17, with Met-12 and Val-16 being the critical residues. Therefore, it is hypothesized that Met-12 and Val-16 directly interacts with some cytoplasmic domain of Kv4 subunits to oppose the effects of KChIPs and thereby slow recovery and regulate SSI. Furthermore, heterologous expression studies using different KChIP variants indicate that the functional effects associated with DPP6K are highly dependent on the variants of KChIP subunits present, suggesting that the precise combination of KChIP and DPLP N-terminal variants expressed by neurons play a critical role in determining the functional properties of I_SA_ and therefore AP firing properties.

## THE Kv4-KChIP-DPLP TERNARY COMPLEX AND DIVERSITY OF NEURONAL I_SA_

### THE TERNARY COMPLEX AND SURFACE TRAFFICKING

Most KChIPs and all DPLPs subunits increase surface trafficking and functional expression of Kv4 channels. The apparent redundancy of this functional effect among the two classes of auxiliary subunits has been addressed in detail by knockout or RNAi studies in native neurons where one class of subunit is deleted. KChIP3-specific knockout has no effect on Kv4.2 protein level (Alexander et al., 2009), and in hippocampal neurons, KChIP2 deletion (*Kcnip2*^-/-^) diminished I_SA_ only moderately (Wang et al., 2013). In cortical pyramidal neurons from mice, the results show that RNAi suppression of individual KChIPs (KChIP2, KChIP3, and KChIP4) can be compensated by the reciprocal increases in the expression of other KChIPs (Norris et al., 2010). In dealing with compensation, simultaneous suppression of KChIP2–4 by miRNA reportedly eliminated a significant fraction of the current (~50%) without altering the voltage dependence of activation and the kinetics of inactivation. On the other hand, targeted deletion of Kv4.2 in neurons produces dramatic reduction in the level of KChIP proteins that precisely tracks the loss of Kv4.2 protein normally found a given region (Menegola and Trimmer, 2006; Nadin and Pfaffinger, 2010; Norris et al., 2010). These findings suggest that perhaps the binding of Kv4 subunits by KChIP proteins inhibits or suppresses degradation by the ubiquitin-proteosome pathway (Jang et al., 2011).

Compared to KChIPs, DPLPs clearly play a central role in the surface expression of I_SA_ channels. In contrast to the moderate reduction associated with the inhibition of KChIP expression, suppression of DPP6 by RNAi dramatically decreases the protein levels (90%) of Kv4.2, Kv4.3, and KChIP3 in CG cells and hippocampal neurons (Nadin and Pfaffinger, 2010). Genetic knockout of DPP6 produces a marked reduction of Kv4.2 and KChIP2/KChIP4 proteins in hippocampus, particularly in the distal dendrites (Sun et al., 2011). On the contrary, targeted deletion of Kv4.2 and/or Kv4.3 in neurons has little to no effect on the DPP6 proteins levels (Nadin and Pfaffinger, 2010; Foeger et al., 2012). Together, these results suggest a model of ternary channel complex trafficking where KChIP stability is dependent upon Kv4 protein, Kv4 protein stability is dependent upon DPLP binding, and DPLPs can exist in a neuron independently of the I_SA_ channel complex.

What mechanism underlies this dependence of surface expression on DPLP? In other words, why would Kv4.2 channels, despite KChIP’s innate ability to promote surface expression by sequestering Kv4’s N-terminal ER retention motif, continue to be prevented from reaching the cell surface? The answer may lie with the ability of KChIP2x, KChIP3x, and KChIP4a to mediate ER retention and potentially targeted for degradation (Jerng and Pfaffinger, 2008), and more experiments will be required to investigate the neuronal machinery involved in the quality control of I_SA_ channels arriving on the cell surface.

A final important question is how are these three proteins regulated to determine the level of I_SA _produced in a given neuron. Based on a variety of analyses, it seems that all surface I_SA_ channels in neurons minimally contain all three subunit types. In CG cells, rescue of DPP6 knockdown by over-expression of RNAi-insensitive DPP6 constructs returns I_SA_ back to normal levels, suggesting that DPP6 expression level is normally high enough to drive all available I_SA_ channels to the cell surface (Nadin and Pfaffinger, 2010). On the other hand, when Kv4.2 has been over-expressed in neuron, the amplitude of I_SA_ undergoes dramatic increases, suggesting that sufficient KChIP and DPP6 subunits are normally expressed in neurons to accommodate the additional Kv4.2 subunits being produced (Johns et al., 1997; Lauver et al., 2006). It therefore seems likely that in most neurons, the overall level of I_SA_ may be normally limited by the level of Kv4 subunit expression.

### THE TERNARY COMPLEX AND FUNCTIONAL PROPERTIES

In native cells, the association of KChIP and DPLP subunits with Kv4 also has important consequence for channel functional properties. Studies using heterologous expression systems suggest that, for most channel properties, the combined modulation of Kv4 channels by KChIP and DPLP are usually the summed effects of the individual subunits. For example, KChIPs shift the SSI curve rightward, whereas DPLPs shift it leftward. Thus, a channel containing both KChIP and DPLP will have a SSI curve intermediate between either subunit alone. On the other hand, since both KChIP and DPLPs accelerate recovery from inactivation, the ternary complex exhibits recovery from inactivation faster than with either subunit alone (Jerng et al., 2005).

Examining the specific KChIP and DPLP variants being expressed in different neuronal populations explains how the interaction of KChIP and DPLP modulatory effects shape distinctive I_SA _in these different neurons. For example, DPP10a and DPP6a distinctly produce fast N-type inactivation by blocking the internal pore with their N-terminal domains (**Figure 5B**). In the ternary complex, the fast inactivation associated with these unique subunits dominates and characteristically produces inactivation that accelerates with increasing depolarization and is unaltered by the presence of KChIPs (Jerng et al., 2005, 2009; Maffie et al., 2009). In contrast, in the presence of other DPLP subunits, the binding of KChIP to the Kv4 N-terminus eliminates endogenous N-type inactivation of Kv4 channels and reveals the underlying CSI, which slows with increasing depolarization.

#### Cortical pyramidal neurons

Cortical pyramidal neurons express I_SA_ with rapid inactivation kinetics (τ = ~10 ms) relatively independent of voltage at positive membrane potentials (Zhou and Hablitz, 1996; Korngreen and Sakmann, 2000). A combination of Kv4.2, Kv4.3, KChIP2–4, DPP6S, and DPP10a transcripts is expressed in these neurons (**Figures 3A,B**), and a recent report indicates that miRNA-mediated simultaneous knockout of KChIP2–4 in Kv1.4^-/-^ cortical pyramidal neurons produced a significant reduction in I_SA_ peak current with no observed changes in the inactivation kinetics (Norris et al., 2010). Since the KChIP component does not significantly influence inactivation properties and the phenotypically dominant DPP10a variant is expressed at high levels, co-expression of Kv4.2, KChIP3a, and DPP10a in heterologous cells was sufficient to generate an A-type current with inactivation kinetics and voltage dependence very similar to that of the cortical I_SA_ (Jerng et al., 2007). Co-expression of Kv4.2 and KChIP3a with the other DPLP present, DPP6S, did not produce cortical I_SA_-like fast inactivation. These results suggest that DPP10a-mediated N-type inactivation is critically important to the inactivation kinetics of cortical I_SA_.

#### Superior colliculus neurons

I_SA_ among superior colliculus neurons exhibits varying inactivation rates and voltage-dependent properties (Saito and Isa, 2000). The time constant of inactivation ranges between 12 and 65 ms, but the voltage dependence of inactivation kinetics at positive potential remains monotonic and suggests DPP10a- or DPP6a-mediated N-type inactivation. In situ hybridization studies indicate that DPP6a is highly expressed in superior colliculus, but in addition DPP6S and DPP6K are also expressed at moderate levels (Nadal et al., 2006; Maffie et al., 2009; Jerng and Pfaffinger, 2012). These results suggest that varying ratios between DPP6a and other DPP6 variants in individual neurons may produce the observed variable functional properties, and the differences are physiologically significant since different I_SA_ properties are associated with different firing properties among the superior colliculus neurons (Saito and Isa, 2000).

#### Cerebellar granule cells

**FIGURE 7:**
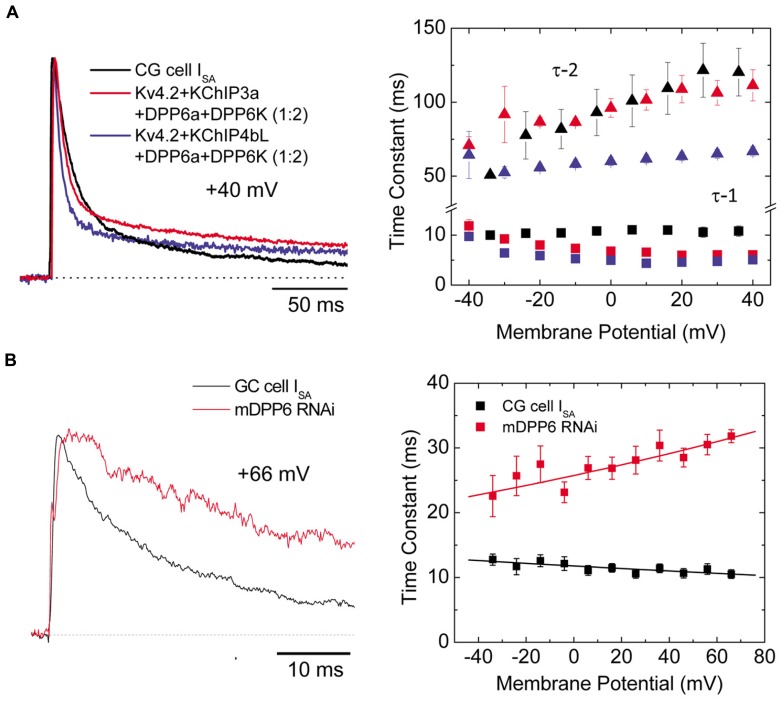
**Ternary channel complex consisting of Kv4.2, KChIP3a, DPP6a, and DPP6K express A-type current similar to I_SA_ from CG cells (Adapted from Nadin and Pfaffinger, 2010; Jerng and Pfaffinger, 2012). (A)** Comparison between native I_SA _from CG cells and reconstituted currents expressed by Kv4.2, KChIP, and DPP6 subunits. Based on real-time RT-PCR results, the ratio between DPP6a and DPP6K was determined to be 1-to-2 (Jerng and Pfaffinger, 2012). Native and reconstituted current traces elicited by +40 mV depolarizations are quite similar (left panel). The incorporation of KChIP3a verses KChIP4bL produces negligible effect on overall current waveforms. The overall inactivation is best described by the sum of two exponential components, and the two corresponding time constants (τ -1, τ -2) have different dependence on membrane potential (right panel). **(B)** In the absence of DPP6, I_SA_ inactivation significantly slows (left panel). The loss of DPP6 altered the inactivation-voltage profile (right panel). Control I_SA_ shows inactivation with time constant relatively insensitive to depolarization. After DPP6 knockdown by RNAi, inactivation time constant slows with increasing depolarization. Note that panels **A** and **B** have separate legends.

During prolonged depolarization, the I_SA_ from CG cells features a prominent biphasic inactivation, where the fast inactivation is voltage-independent and the slow inactivation becomes slower with increasing potential (Zegarra-Moran and Moran, 1994). As a result, in contrast with I_SA_ from cortical and superior colliculus neurons, I_SA _from CG cells inactivates overall more slowly at higher membrane potential (Amarillo et al., 2008). *In situ* hybridization and RT-PCR studies of the I_SA_ subunits present show that CG cells express a mixture of Kv4.2, Kv4.3, DPP6, and KChIP3, with some contribution from various KChIP4 variants (**Figure 3**). The levels of Kv4.2 and Kv4.3 transcripts are not uniform among CG cell layer, since they exhibit opposite anterior–posterior gradients (Amarillo et al., 2008). Among the DPP6 variants, DPP6K and DPP6a comprise the vast majority of DPP6 expressed in CG cells, with DPP6K being the predominant variant (Jerng and Pfaffinger, 2012). DPP6K appears to be important to the Kv4.2 channel complexes since it shares the same anterior–posterior gradient of expression in the CG cell layer (Nadal et al., 2006). Co-expression of Kv4.2 and KChIP with DPP6K and DPP6a at the experimentally derived ratio (2:1) in *Xenopus* oocytes reconstitutes a transient current that highly resembles the native I_SA_ in CG cells (**Figure 7A**, *left panel*; Jerng and Pfaffinger, 2012). Like the native I_SA_, the reconstituted current inactivated with two components of inactivation characterized by different voltage dependent properties (**Figure 7A**, right panel). In another co-expression study, Maffie et al. (2009) also generated the best match between the inactivation kinetics of reconstituted and native currents using DPP6S and DPP6a at a 2-to-1 ratio. The contribution of DPP6a-conferred N-type inactivation to native I_SA_ inactivation was further addressed by RNAi experiments conducted on CG cells. Suppression of DPP6 expression by RNAi in mouse CG cells markedly slowed I_SA_ inactivation kinetics and altered its voltage dependence (**Figure 7B**; Nadin and Pfaffinger, 2010), suggesting that DPP6a-conferred N-type inactivation contribute significantly to the inactivation of I_SA_. Furthermore, fast N-type inactivation is restored when rat DPP6a rescue protein is over-expressed in mouse CG cells treated with DPP6 RNAi. Therefore, significant dilution of DPP6a by DPP6K and other DPP6 variants very likely results in the bi-phasic inactivation of I_SA_ in CG cells, rather than other factors such as a loss of N-type inactivation associated with DPP6a.

#### Hippocampal neurons

Hippocampal I_SA_ displays inactivation kinetics that slows with increasing depolarization (Hoffman et al., 1997), and this voltage dependence of inactivation appears consistent with the finding that neither DPP10a nor DPP6a is highly expressed in these neurons (Jerng et al., 2007; Jerng and Pfaffinger, 2012). Overall, knockout experiments show that KChIP and DPP6 play an important role in suppressing excitability in hippocampal neurons. In hippocampal pyramidal neurons, the deletion of KChIP2 gene (*Kcnip2*^-/-^) moderately reduced peak I_SA _and shifted the V_1/2 _of SSI to more hyperpolarized potentials (Wang et al., 2013). As a result, hippocampal neurons with the KChIP2 gene knocked out show increased excitability; in fact, the average spontaneous firing rate increased 10-fold. In hippocampal interneurons, suppression of KChIP1 by siRNA moderately reduced peak I_SA_ and slowdown recovery from inactivation (Bourdeau et al., 2011). As a result, firing frequency during suprathreshold depolarizations was increased. Meanwhile, hippocampal CA1 neurons with DPP6 knockout show a reduction of I_SA_ in distal dendrites (Sun et al., 2011). In contrast with the KChIP2 knockout, the decreased I_SA_ is associated with depolarizing shift in the voltage-dependence of activation, and these effects result in more backpropagation of action potentials, increased excitability of dendrites, enhanced electrical activities, and increased induction of synaptic LTP.

## KChIPs AND DPLPs: BEYOND THE I_SA_ CHANNEL

Kv channel-interacting proteins and DPLPs, as constitutive components of the I_SA_ channel complex, contribute to neuronal excitability by tuning the properties of I_SA_. However, recent studies suggest that these proteins are more than just auxiliary subunits of I_SA_ channels; KChIPs and DPLPs directly modulate other ion channels or receptors, or act as intermediaries between I_SA_ channels and other membrane proteins.

### KChIPs AND Cav CHANNEL (T-Type, L-Type)

**FIGURE 8:**
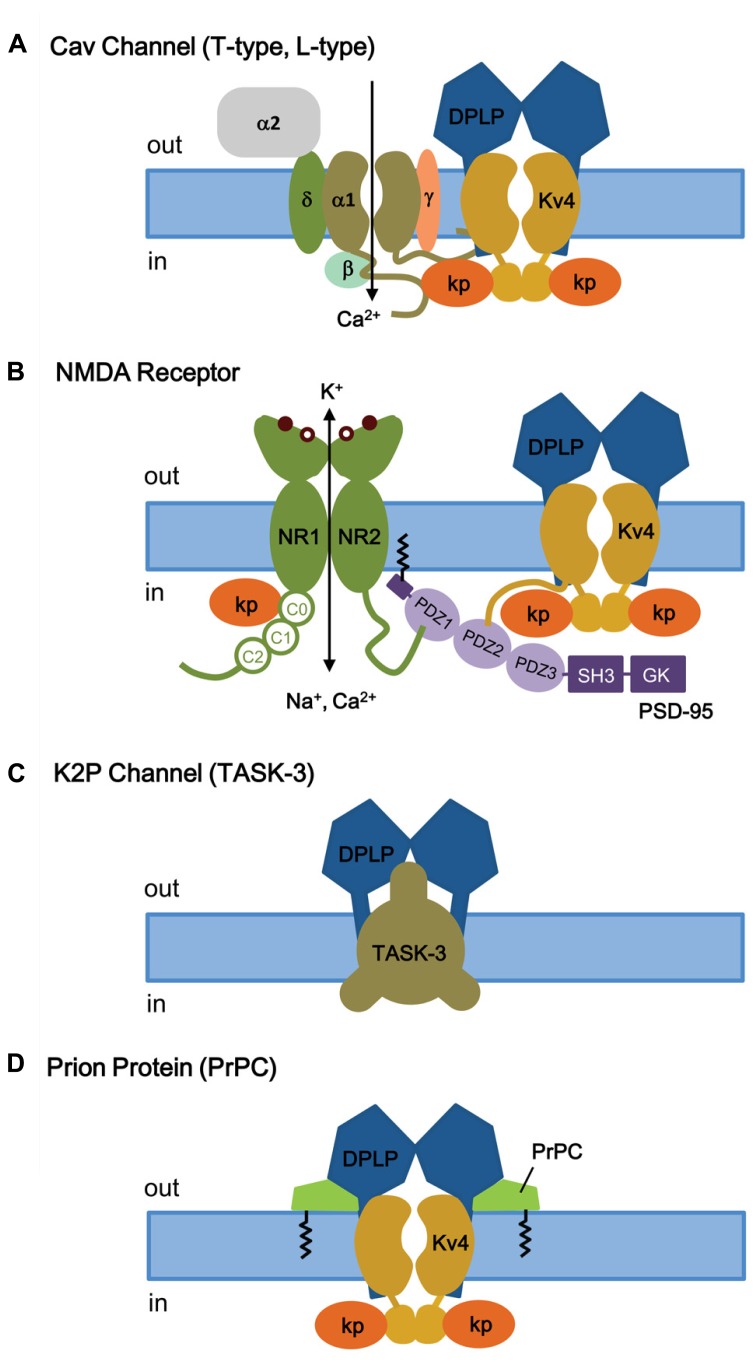
**KChIP and DPLP are also involved in binding and regulating other channels and receptors. (A)** T- and L-type Cav channels. Depolarization-activated Cav channels, like I_SA_ channels, are multi-protein complexes. The cytoplasmic N- and C-termini of Cav channels have been shown to interact with the I_SA_ channel complex, via Kv4 or KChIP subunits. **(B)** NMDA receptor. The NMDA receptors are opened by 2 simultaneous events: activation by extracellular ligand binding and depolarization-mediated relief of block by Mg^2^^+^ (coincidence detector). Both NMDA receptor and I_SA_ channels are localized post-synoptically, and KChIPs have been shown to regulate NMDA receptor activities. **(C)** TASK-3 two-pore K^+^ channels. DPP6 co-assembles with TASK-3 channels in the dendritic membrane to regulate membrane excitability. **(D)** Prion proteins (PrPC) bind to DPP6, thereby regulating I_SA_ expression and gating. Kp, KChIP; NR1, glycine-binding subunit; NR2, glutamate-binding subunit; SH3, SRC homology 3 domain; GK, guanylate kinase.

Voltage-gated Ca^2^^+^ (Cav) channels are activated by membrane depolarization, and their opening allows Ca^2^^+^ to enter the cell and initiate intracellular Ca^2^^+^ signaling events. In the brain, the T-type Ca^2^^+^ channel and Kv4 channels are widely expressed with high expression in the somatodendritic regions of neurons (McKay et al., 2006), and they both operate at the subthreshold range of membrane potential. Anderson et al. (2010) showed that in cerebellar stellate cells the Ca^2^^+^ influx mediated by T-type Cav channels modulates I_SA_, by producing a depolarizing shift in the SSI of I_SA_ by approximately 10 mV and modulating I_SA_ window current and spike firing (Anderson et al., 2010). This effect of Ca^2^^+^ on SSI is specific and has no effect on the voltage dependence of activation, the kinetics of inactivation and recovery, or the I_SA_ current density. The Ca^2^^+^ sensitivity of the Kv4 complex is specifically produced by KChIP3 and not by KChIP1 or KChIP2, indicating a critical role for KChIP3 in the modulation of I_SA_ in CG cells. In addition, there must be a close proximity between Cav channels and I_SA_ channels, since Cav3.2 and Cav3.3 co-immunoprecipitate with Kv4.2 and KChIP3 proteins from rat brain homogenates, including those from cerebellum, hippocampus, and neocortex (**Figure 8A**; Anderson et al., 2010). This co-immunoprecipitation depends on the C-terminal regions of Cav3.2 and Cav3.3, suggesting that the Cav3 C-terminus interacts with the I_SA_ complex. However, it remains unclear whether the Cav3 C-terminus binds Kv4 or KChIP3 subunits. Physiologically, in the cerebellum the Cav-Kv4 channel complex acts as a Ca^2^^+^ sensor that responds to a decrease in extracellular Ca^2^^+^ by dynamically adjusting stellate cell excitability to maintain inhibitory charge transfer to Purkinje cells (Anderson et al., 2013). The pre-synaptic N-type Cav2.2 channels which are activated by high voltage have no effect on Kv4.2 availability (Anderson et al., 2010), suggesting that only post-synaptic Cav channels have evolved to function alongside KChIP and I_SA_ channel complex.

In cardiac myocytes, it was found that *Kcnip2*^-^^/^^-^ mice have reduced L-type Ca^2^^+^ current (I_Ca,L_) by 28% compared to wild-type myocyte (Thomsen et al., 2009b). L-type Cav channels are also found in the dendrites and dendritic spines of cortical neurons, and recently a Ca^2^^+^-independent regulation of I_Ca,L_ by KChIP2 was found in neurons (Thomsen et al., 2009a). Biochemical analysis shows that there is a direct interaction between KChIP2 and the Cav1.2 alpha-1C subunit N-terminus, and the binding of KChIP2 to the N-terminal inhibitory (NTI) module of alpha-1C augments the I_Ca,L_ current density without increasing protein expression or trafficking to the plasma membrane. More studies will be required to decipher the different Ca^2^^+^ dependence observed between T- and L-type Cav channels, although these studies suggest important ways that different KChIPs may function separately from their direct effects on Kv4 channels.

### KChIPs AND NMDA RECEPTOR

The N-methyl-D-aspartate receptors (NMDA receptors, NMDARs) make up a major class of ionotropic glutamate receptors that, like I_SA_ channels, are involved in controlling synaptic plasticity and memory function (Maren and Baudry, 1995; Asztely and Gustafsson, 1996). The NMDA receptor is a non-specific cation channel located in the postsynaptic membrane that allows Ca^2^^+^ and Na^+^ to pass into the cell while K^+^ flows out, with an equilibrium potential near 0 mV (Dingledine et al., 1999). At rest, the NMDA receptor is blocked by Mg^2^^+^, which can be cleared by depolarization. Since both glutamate binding and postsynaptic depolarization are required for channel opening, the NMDA receptor functions as a “coincidence detector.” The NMDR receptor is a heterotetramer between two NR1 (glycine-binding) and two NR2 (glutamate-binding) subunits (Traynelis et al., 2010; **Figure 8B**). The extracellular ligand-binding domain is attached to the transmembrane core domain that determines conductance, Ca^2^^+^-permeability, and voltage-dependent Mg^2^^+^ block. The cytoplasmic domain contains residues that can be modified by protein kinases and phosphotases as well as residues that interact with structural proteins. The C-terminus of NMDA receptor binds to the postsynaptic density-95 (PSD-95) at a PDZ domain, which anchors itself through S-palmitoylation of an N-terminal domain (**Figure 8B**; Craven et al., 1999; Sturgill et al., 2009).

Like several receptors and Kv1 channels, Kv4.2 subunits bind to PSD-95 via an amino acid sequence motif (VSAL) in its C-terminal tail, allowing channel clustering and promoting localization into lipid rafts (**Figure 8B**; Wong et al., 2002; Wong and Schlichter, 2004). Although the exact detail remains unclear, in addition to its role as a Kv4 auxiliary subunit, KChIP3a can directly bind in a Ca^2^^+^-dependent manner to a region of the NMDAR juxta-transmembrane C-terminus (C0 cassette, amino acids 834–863) of the NR1 subunit, thereby inhibiting NMDAR-mediated current and excitotoxicity (Zhang et al., 2010). As for the region of KChIP3a that binds NMDAR, the first 50 residues as well as the core domain are important for interaction. It appears that, in addition to S-palmitoylation to allow membrane association and localization to lipid rafts, the N-terminal cytoplasmic domain of KChIP3a is involved in association with NMDA receptors. At this time, it is unknown whether KChIP3a, when interacting with NR1 subunits, is associated or unassociated with Kv4. In either case, the KChIP subunit apparently again acts as a Ca^2^^+^ sensor that, with NMDRs, functions as a negative regulator to protect neurons from cytotoxic injury.

### DPP6 AND K2P CHANNEL (Task-3)

Two-pore K^+^ channels (K2P) channels generate a K^+^ leak conductance (I_K(SO)_) and set the resting membrane potential in many neuronal populations. Opening of K2P channels promote hyperpolarization, and inhibition of K2P channels lead to depolarization. TASK-3 channels are members of the K2P channel family that is sensitive to acid and inhalation anesthetics at clinically relevant concentrations and widely expressed in the rodent brain (Talley et al., 2001; Vega-Saenz de Miera et al., 2001; Aller et al., 2005; Aller and Wisden, 2008). At a subcellular level, TASK-3 channel are expressed in the somatodendritic regions of the neuron, much like Kv4 channels (Marinc et al., 2012).

RNAi-mediated suppression of DPP6 expression in CG cells from Kv4.2 knockout mouse reveals that DPP6 is affecting the resting membrane potential by influencing a channel other than Kv4 (Nadin and Pfaffinger, 2013), most likely the K2P channel TASK-3. TASK-3 is the most highly expressed K2P subunit in CG cells, and consistent with TASK-3, the DPP6-modulated resting membrane conductance is sensitive to acidic pH and Zn. In addition, reconstitution and immunoprecipitation studies in CHO-K1 cells show that DPP6 co-immunoprecipitates with TASK-3 and its co-expression increases the TASK-3 current, suggesting that DPP6 interacts with TASK-3 channels (**Figure 8C**).

### DPP6 AND CELLULAR PRION PROTEIN

Cellular prion proteins (PrPC), products of the *PRNP* gene, are cell surface proteins that are widely expressed in various tissues but with the highest levels in neurons in the central nervous system (Bendheim et al., 1992). Under pathological conditions, the alpha-helix-rich PrPC can give rise to the abnormal beta-sheet-rich PrPSc, and the accumulation of PrPSc in the brain produces neurodegenerative disorders known as prion diseases. The transmission and development of prion disease is proposed to occur by a process of homotypic conversion from normal to abnormal prion protein, whereby the endogenous PrPC interacts with an incoming PrPSc or *de novo* PrPSc generated by some unknown post-translational process (Telling et al., 1995; Kaneko et al., 1997).

Currently, the normal biological function of PrPC remains mostly unknown. PrPC knockout studies in mice initially suggest that PrPC is not required for embryonic development or normal neuronal excitability and synaptic transmission in hippocampal CA1 region (Bueler et al., 1992; Lledo et al., 1996). However, subsequent studies suggest that PrPC knockout is linked to abnormalities in motor, cognitive, and emotional functions, along with increased susceptibility to seizures and alterations in excitability and synaptic plasticity (Roesler et al., 1999; Walz et al., 2002; Criado et al., 2005; Prestori et al., 2008). The strongest evidence for PrPC function comes from *in vitro* studies, which suggest that PrPC may be involved in neuriteogenesis, cell adhesion, and binding of neurotrophic factors. Neurons from PrPC knockout mice in culture shows reduced Ca^2^^+^-dependent K^+^ current (Colling et al., 1996; Herms et al., 2001; Mallucci et al., 2002) and reduced cytoplasmic Ca^2^^+^ levels (Herms et al., 2000). The reduction in Ca^2^^+^ levels is proposed to be due to PrPC modulation of L-type Cav channels in CG cells (Korte et al., 2003), which can subsequently affect the Ca-dependent K current.

To further understand the normal physiological role of PrPC, studies were conducted to identify PrPC-interacting proteins by analyzing high-density protein microarrays and by protein cross-linking followed by purification and mass spectrometry (tbTPC; Schmitt-Ulms et al., 2004; Satoh et al., 2009). The tbTPC technique identifies DPP6 as part of the molecular microenvironment of PrPC (Schmitt-Ulms et al., 2004), and just recently Mercer et al. (2013) demonstrated that PrPC co-immunoprecipitates with DPP6 and modulates Kv4.2 channels in a DPP6-dependent manner (**Figure 8D**; Mercer et al., 2013). PrPC modulates the I_SA_ channel complex by increasing peak current amplitude, rightward shifting the voltage dependence of SSI, slowing inactivation kinetics, and accelerating recovery from inactivation. These functional effects overall decrease membrane excitability and susceptibility to seizures and explains why the PrPC knockout alters excitability, synaptic plasticity, and susceptibility to seizures. In addition, since DPP6 also associates with the K2P channel TASK-3, PrPC may also interact with the K2P-DPP6 channel complex.

## THE ROLE OF I_SA_ AND ITS AUXILIARY SUBUNITS IN REGULATING EXCITABILITY

The roles that I_SA _and its regulation play in controlling neuronal excitability is complex and involves the dynamic interplay between I_SA_ and multiple other channel types. The regulation of excitability is the net effect of the strengths of synaptic inputs, the integrative properties of the somatodendritic compartment, and the threshold firing properties of the axon initial segment. I_SA_ directly or indirectly affects all of these steps. Being a K^+^ current, I_SA_ primarily plays a regulatory function, controlling how readily depolarizing currents gain control of the membrane potential and how effectively action potential firing is backpropagated to the synapse to regulate synapse strength. The unique regulatory properties of I_SA_ compared to other K^+^ currents involve the voltage range over which the channel gates, the kinetics of the channel’s gating, its subcellular localization in the neuron, as well as the sensitivity of I_SA_ to specific post-translational regulatory phenomena. A few of the ways in which rapid activation of I_SA_ at subthreshold potentials directly regulates excitability includes: (1) suppression of AMPA responses (Schoppa and Westbrook, 1999), (2) suppression of back propagating action potentials (Hoffman et al., 1997), and (3) delayed firing in response to current injections (Nadin and Pfaffinger, 2010). Because I_SA_ inactivates rapidly, these regulatory responses are all time limited and rapidly lost unless reset by a subsequent hyperpolarization. This timing dependence for I_SA _regulation is an additional coincidence detection mechanism that operates in parallel with the well described coincidence detection role for Mg^2^^+^ block of NMDA receptors (Schrader et al., 2002). For example, in hippocampal pyramidal neurons, incoming AMPA response depolarize dendrites enough to drive I_SA_ inactivation (Watanabe et al., 2002). If the drive is enough to initiate the firing of an action potential, the backpropagation of this spike will be suppressed by I_SA_ in regions of the dendritic tree that were not previously depolarized (Hoffman et al., 1997). However, in the region of the active synapses, the inactivation of I_SA_ will allow a large spike to propagate resulting in a large Ca^2^^+^ flux through NMDA receptors (Johnston et al., 2003). The sensitivity of this response is modified during LTP protocols by phosporylation of I_SA_ and thus is a tunable mechanism capable of refining the assignment of Hebbian synaptic strengthening to the synapses in dendritic regions that drove action potential firing (Frick et al., 2004).

Because I_SA_ gating properties are strongly regulated by auxiliary subunit proteins, excitability and coincidence detection in different neuronal types are likely to be extensively tuned by the I_SA_ auxiliary subunit protein variants that are being expressed. The interaction of KChIPs and DPLPs with other channels and receptors will also modulate the overall excitability properties of the neuron. As an illustration of the important role of non-Kv4 proteins in excitability, knockout of Kv4.2 effectively eliminates I_SA_ from hippocampal neuronal dendrites, but compensatory changes in glutamatergic transmission and inhibitory connections occur and likely compensate for much of the impact of this knockout (Andrasfalvy et al., 2008; Jung et al., 2008; Kim and Hoffman, 2012; Kaufmann et al., 2013). It is likely that many of the compensatory effects noted in ion channel knockout studies are due to the integrative effects of auxiliary subunit protein (Lin et al., 2013; Wang et al., 2013). In addition, I_SA_ is reported to be regulated by auxiliary subunits proteins for other channels, further implicating auxiliary protein in the integrative regulation of excitability. For now, however, the specific manner in which these integrative interactions work together to control neuronal functional phenotypes is not well understood. A key question for future studies is the extent to which compensatory remodeling is being driven at the systems level or is the direct result of signals being generated by loss of ion channel complexes and the protein-protein interactions that these channel complexes normally participate in.

## SUMMARY AND CONCLUSION

With the physiological role of I_SA_ firmly established and the central role of Kv4 subunits in I_SA_ generation clearly recognized, in recently years the attention has been focused on the modulatory roles of Kv4 auxiliary subunits and their contribution to the tuning of I_SA_ biophysical properties, the creation of functional diversity in neurons, the regulated trafficking and subcellular localization of I_SA _channels, and the expanding interaction between I_SA_ and other signal transduction pathways within neurons. What we have learned from the detailed molecular understanding of I_SA_ channels is that, at a minimal level, I_SA_ is the product of Kv4 channels and a complex network of interactions, between Kv4 and a “tool kit” of auxiliary subunits that also senses and feeds back modulatory signals to alter the I_SA_. This modulatory signal may include the environment redox state that specifically regulates fast inactivation, or the local Ca^2^^+^ concentration that alters I_SA_ function through KChIP. Furthermore, we have also learned that auxiliary subunits play a critical role in the proper assembly and trafficking of I_SA_ channels to the cell surface and the recruitment of these channels to lipid rafts and then post-synaptic densities.

Lastly, as part of a greater network, KChIP and DPLPs have been proposed to have functions independent of their helper roles on I_SA_, and substantially more study is required to further explore define their roles in neuronal physiology. Just recently, Lin et al. (2013) show that DPP6 interacts with a filopodia-associated myosin and fibronectin in the extracellular matrix, and DPP6 is important to cell adhesion and motility that ultimately impact synaptic development and function. Because of their potential multi-faceted roles, it is perhaps not surprising that KChIPs and DPLPs are increasingly linked to various diseases and disorders, including autism spectrum disorder and schizophrenia (**Table 2**). Only with better understanding of functional roles of these auxiliary subunits can we begin to further unravel the mystery behind these disorders and develop targeted treatments.

**Table 2:** Genetic association between I_**SA**_ subunits and disease/disorders.

Subunit	Phenotype	Subject	Conclusion and analysis method	Reference
Kv4.2	Temporal lobe epilepsy	Human-Japanese	Yes: electrophysiology	Singh et al. (2006)
KChIP	Ventricular tachycardia	Mouse	Yes: KChIP2 knockout	Kuo et al. (2001)
	Pain	Mouse	Yes: KChIP3 knockout	Cheng et al. (2002)
DPP6	Amyotrophic lateral sclerosis	Human-multi-ethnic	Inconsistent: GWAS analysis SNP analysis CNV association	Cronin et al. (2008), van Es et al. (2008), Chio et al. (2009), Li et al. (2009), Blauw et al. (2010), Fogh et al. (2011)
	Progressive multiple sclerosis	Human-Italian	Yes: SNP analysis	Brambilla et al. (2012)
	Tardive dyskinesia schizophrenia	Human-Japanese	Yes: GWAS analysis	Tanaka et al. (2013)
	Autism spectrum disorder	Human-multi-ethnic	Yes: ID mutations CNV association	Marshall et al. (2008), Noor et al. (2010)
	Microcephaly	Human-Chinese	Yes: CNV association	Liao et al. (2013)
DPP10	Asthma	Human-multi-ethnic	Yes: SNP association GWAS analysis Positional cloning	Allen et al. (2003), Zhou et al. (2009), Wu et al. (2010), Torgerson et al. (2012)
	Autism spectrum disorder	Human-mixed	Yes: CNV analysis	Girirajan et al. (2013)
	Bipolar disorder schizophrenia	Human-Norwegian	Yes: GWAS analysis	Djurovic et al. (2010)

*
GWAS, genome-wide association studies; CNV, copy-number variants; SNP, single-nucleotide polymorphism.
*

## Conflict of Interest Statement

The authors declare that the research was conducted in the absence of any commercial or financial relationships that could be construed as a potential conflict of interest.
